# SveDem, the Swedish Dementia Registry – A Tool for Improving the Quality of Diagnostics, Treatment and Care of Dementia Patients in Clinical Practice

**DOI:** 10.1371/journal.pone.0116538

**Published:** 2015-02-19

**Authors:** Dorota Religa, Seyed-Mohammad Fereshtehnejad, Pavla Cermakova, Ann-Katrin Edlund, Sara Garcia-Ptacek, Nicklas Granqvist, Anne Hallbäck, Kerstin Kåwe, Bahman Farahmand, Lena Kilander, Ulla-Britt Mattsson, Katarina Nägga, Peter Nordström, Helle Wijk, Anders Wimo, Bengt Winblad, Maria Eriksdotter

**Affiliations:** 1 Karolinska Institutet, Department of Neurobiology, Care Sciences and Society, Center for Alzheimer Research, Division for Neurogeriatrics, Huddinge, Sweden; 2 Geriatric Clinic, Karolinska University Hospital, Stockholm, Sweden; 3 Karolinska Institutet, Department of Neurobiology, Care Sciences and Society, Center for Alzheimer Research, Division of Clinical Geriatrics, Stockholm, Sweden; 4 Trädgårdstorgets Primary Care Unit, Linköping, Sweden; 5 Municipality of Norrtälje, Norrtälje, Sweden; 6 Central hospital, Karlstad, Sweden; 7 Department of Public Health and Caring Sciences/Geriatrics, Uppsala University, Uppsala, Sweden; 8 Neuropsychiatric clinic, Sahlgrenska University Hospital, Gothenburg, Sweden; 9 Clinical Memory Research Unit, Department of Clinical Sciences Malmö, Lund University, Malmö, Sweden; 10 Department of Community Medicine and Rehabilitation, Geriatric Medicine, Umeå University, Umeå, Sweden; 11 Sahlgrenska Academy, Institute of Health and Care Sciences at Gothenburg University, Gothenburg, Sweden; 12 Centre for Research & Development, Uppsala University/County Council of Gävleborg, Gävle, Sweden; University of Glasgow, UNITED KINGDOM

## Abstract

**Background:**

The Swedish Dementia Registry (SveDem) was developed with the aim to improve the quality of diagnostic work-up, treatment and care of patients with dementia disorders in Sweden.

**Methods:**

SveDem is an internet based quality registry where several indicators can be followed over time. It includes information about the diagnostic work-up, medical treatment and community support (www.svedem.se). The patients are diagnosed and followed-up yearly in specialist units, primary care centres or in nursing homes.

**Results:**

The database was initiated in May 2007 and covers almost all of Sweden. There were 28 722 patients registered with a mean age of 79.3 years during 2007–2012. Each participating unit obtains continuous online statistics from its own registrations and they can be compared with regional and national data. A report from SveDem is published yearly to inform medical and care professionals as well as political and administrative decision-makers about the current quality of diagnostics, treatment and care of patients with dementia disorders in Sweden.

**Conclusion:**

SveDem provides knowledge about current dementia care in Sweden and serves as a framework for ensuring the quality of diagnostics, treatment and care across the country. It also reflects changes in quality dementia care over time. Data from SveDem can be used to further develop the national guidelines for dementia and to generate new research hypotheses.

## Introduction

The number of patients suffering from dementia is increasing, mainly due to the higher proportion of the elderly population [1]. In Sweden, it is estimated that 150 000 individuals suffer from dementia and two thirds of them have Alzheimer´s disease (AD) [2]. The Swedish Dementia Registry (SveDem), a national quality registry for patients with dementia disorders, was initiated to achieve dementia care of similar and high quality for the whole country.

Sweden was one of the first countries in the world to start using quality registries in health and medical services with the main aim to improve the quality of care and reduce regional differences. Registries are important tools for the follow-up of clinical guidelines. They contain individualized data concerning symptoms, medical interventions and outcomes after treatment [3]. Today, there are about 100 different quality registries in Sweden [3] and four competence centers that serve them. They all receive partial funding from the government. One of the oldest examples is the Swedish National Hip Arthroplasty Register, which was started in 1979 [4]. There was a need to create a similar quality database for patients suffering from different dementia disorders, therefore SveDem was established on the 1st of May 2007. Specialist (memory clinics) and primary care units could be affiliated from the beginning and nursing homes became affiliated in 2012. SveDem aims to follow the patients through the chain of care provided by specialist, primary care and nursing home units. Patients are registered by the date when dementia diagnosis is established. Individuals with mild cognitive impairment are not registered in SveDem as this condition was perceived as too vague to be included. Every patient with the diagnosis of dementia should have an annual follow-up to ensure good care. In 2010, the Swedish Board of Welfare published national guidelines of dementia and presented seven clinical indicators which can be followed-up in SveDem.

## Materials and Methods

### Data collection

Data on patients newly diagnosed with dementia is entered into the web-based registry. Information about age, sex, heredity, body mass index (BMI), cognitive evaluation using Mini Mental State Examination (MMSE) score [5], content of diagnostic work-up, type of dementia disorder, pharmacological and non-pharmacological treatment and support for the patient from the county and municipality as well as standard demographic information is registered (Table 1). Data from a yearly follow-up (including diagnosis, MMSE score, pharmacological treatment and received support from the county and municipality) is also recorded.

**Table 1:** List of variables and characteristics recorded in SveDem.

Variable	Type	Value/Unit
Social security number	Numeric	Number
Date of Registration	Date	Date
Time Needed for Diagnosis	Numeric	Days
Sex	Nominal	Female/Male
Age	Numeric	Year
Living Condition	Nominal	Own home/Nursing house/Don’t know; Alone/with someone/Don’t know
Day Care	Nominal	Yes/No/Don’t know
Home Care	Nominal	Yes/No/Don’t know
Family History of Dementia (First degree, Second degree)	Nominal	Yes/No/Don’t know
BMI (Height, Weight)	Numeric	Kg/m^2
Type of Dementia	Nominal	EOAD/LOAD/Mixed AD/Vascular AD/DLB/FTD/PDD/USD/Others
Diagnostic Work-up *(Blood test, clock-test, CT, MRI, LP, PET/SPECT, EEG, Advanced cognitive testing, Assessment by occupational therapist, assessment by physiotherapist, assessment by speech therapist)*	Nominal	Yes/No/Don’t know
Total number of Diagnostic Tests	Numeric	Number
MMSE Score	Numeric	Score
Medication (ChEI, NMDA-Antagonist, Antidepressants, Antipsychotics, Anxiolytics, Hypnotics, Cardiovascular drugs)	Nominal	Yes/No/Don’t know
Possession of Driving License	Nominal	Yes/No/Don’t know
Possession of Weapon License	Nominal	Yes/No/Don’t know
Total Number of Drugs	Numeric	Number
Death	Nominal	Yes/No
Time to Death	Numeric	Months

BMI—body mass index, CT—computed tomography, MRI—magnetic resonance imaging, LP—lumbar puncture, PET—positron emission tomography, SPECT—single photon emission computed tomography, EEG—electroencephalography, MMSE—mini mental state examination, ChEI—cholinesterase inhibitors, NMDA—N-methyl-D-aspartate, EOAD—early onset Alzheimer´s disease, LOAD—late onset Alzheimer´s disease, AD—Alzheimer´s disease, DLB—dementia with Lewy bodies, FTD—frontotemporal dementia, PDD—Parkison´s disease dementia, USD—unspecified

Dementia diagnoses are coded as AD, vascular dementia (VaD), mixed dementia, dementia with Lewy bodies (DLB), frontotemporal dementia (FTD), Parkinson´s disease dementia (PDD), unspecified dementia (where specific dementia diagnosis is not ascertained) and other dementia types (grouping miscellaneous dementia disorders such as corticobasal degeneration or alcohol related dementias). In Sweden, dementia disorders are clinically diagnosed according to the 10^th^ revision of the International Classification of Diseases (ICD-10) [6]. In addition, the McKeith criteria [7] are used for DLB, the Lund-Manchester criteria [8] for FTD and the Movement Disorder Society Task Force criteria [9] for PDD.

Should a patient move to a nursing home, a separate set of specific variables focussing on indicators of nursing care is collected (S1 Table). The data is entered by a staff member (often a nurse or a physician) at the units affiliated with SveDem. A local coordinator for the unit is given a password to enter the registry and can manage the data on the patients cared for at their unit. Descriptive statistics on patients from each unit are available on-line and can be compared with data from other units in the same region as well as with the data from all the SveDem units in Sweden.

### Ethics and legal issues

Quality registries in Sweden are considered as an important part of the development and improvement of health and social care. Each patient has to be informed about the registration and has a right to decline participation. A written consent is not required, however each patient has the right to obtain a copy of the information that is registered if requested. The patient has the right to have their data removed from the registry. An ethical approval from a regional ethics committee for each research project where SveDem data will be used is needed. Ethical permission for this study was obtained from the regional human ethics committee of Stockholm (#2009/209–31). The data is recorded based on each individual´s social security number. A unique number is assigned to all patients. A file linking the personal number, name and identifier is safely stored and managed by Uppsala Clinical Research Centre (www.ucr.se).

### Organization

**Fig 1:**
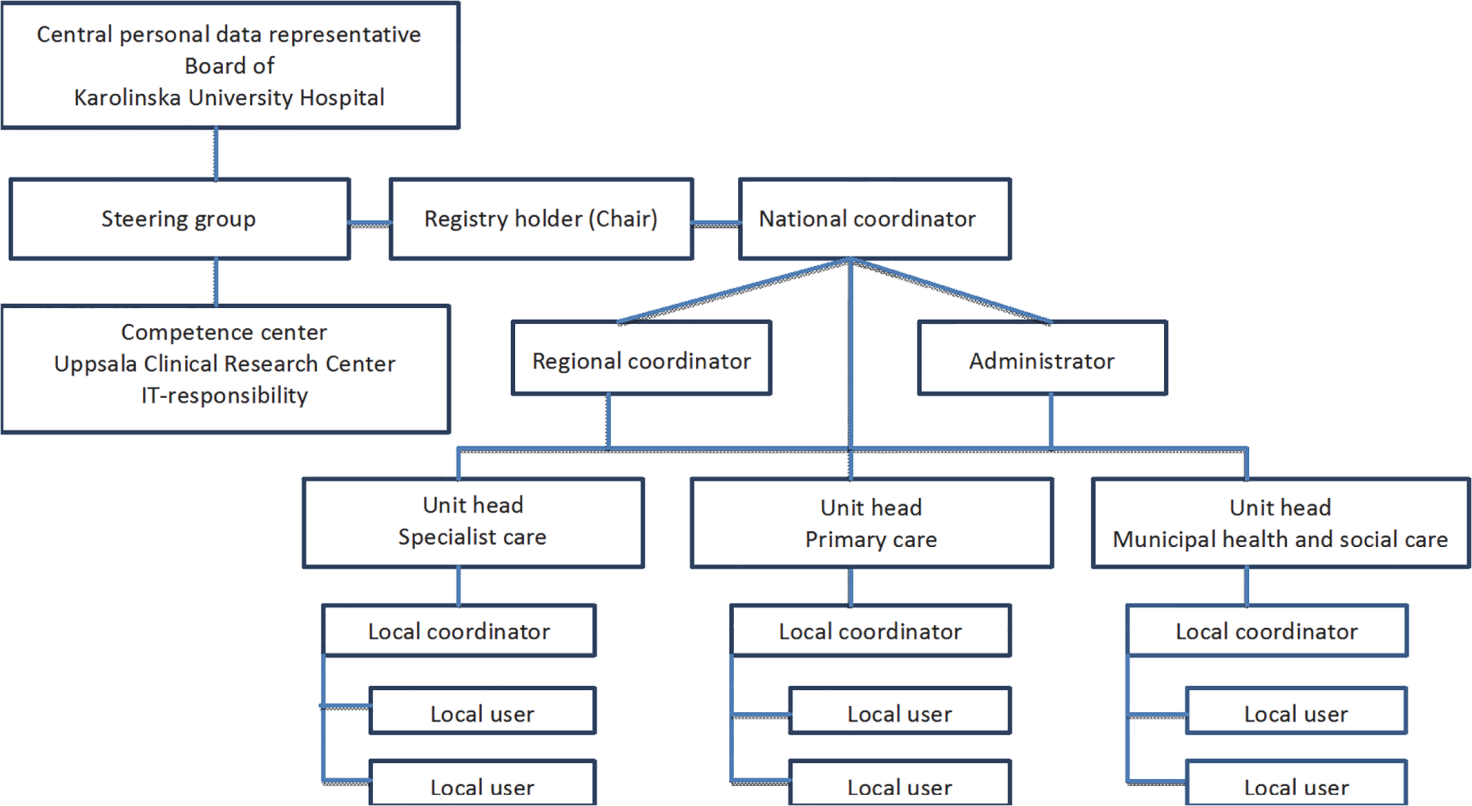
Organisational structure of SveDem.

Although SveDem is a national database, Karolinska University Hospital has the overall responsibility for the data (CPUA). SveDem is governed by a steering committee consisting of representatives from several healthcare professions such as physicians specialized in geriatric medicine, family medicine or psychiatry; nurses, occupational therapists and researchers. SveDem is headed by the registry holder who together with the national coordinator has the responsibility for the everyday functioning of the registry. A fulltime administrator is also employed. Regional coordinators are employed to implement SveDem throughout the country. Uppsala Clinical Research Centre is responsible for the development of the database online, its technical support and data safety. When needed, consultancy competence in epidemiology and statistics is purchased. When a unit is affiliated to SveDem, an agreement between the registry holder and the head of the unit is signed. Each participating unit obtains continuous descriptive statistics from its own registrations online and can compare them with regional and national data. Fig. 1 illustrates an organisational plan of SveDem.

A report from SveDem is published yearly to inform medical and care professionals as well as political and administrative decision-makers about the current quality of diagnostics, treatment and care of patients with dementia disorders in Sweden. SveDem is financed by the Swedish Association of Local Authorities and Regions and the Swedish Brain Power network. SveDem has chosen not to have sponsors from the pharmaceutical industry.

### Data quality

Monitoring is performed by a research nurse who visits units all over the country and verifies if the data in SveDem corresponds to the original data in patients´ medical records. For questions one can call on weekdays between 8 am and 5 pm. Furthermore, 24 hour technical support is available.

## Results

### Characteristics of patients

During the five-year period between 1/5/2007 and 31/12/2012, a total number of 28 722 newly diagnosed dementia patients were registered in SveDem. The population consisted of 16 994 (59.2%) female and 11 728 (40.8%) male dementia patients. The age range of the patients was 27 to 103 years with a mean of 79.3 (SD = 8.0) years. The age-specific sex frequency was higher in female patients and the most common age at diagnosis was 83 for females and 82 for males. The mean MMSE score was 21.1 (SD = 5.1) in the whole population. The majority of the patients had either mild (32.4%; MMSE 20–30 points) or moderate (36.3%; MMSE 10 to <20 points) cognitive impairment at the time when dementia was diagnosed. The majority of the patients lived at home when diagnosed. Other baseline characteristics including living conditions and care utilization are summarized in Table 2.

**Table 2:** Characteristics of the whole population of dementia patients registered in SveDem during 2007–2012 (n = 28722).

Characteristic	Value
Sex-Female n (%)	16994 (59.2%)
Age (yr) mean (SD)	79.3 (8.0)
MMSE score mean (SD)	21.1 (5.1)
Residence at own home n (%)	25492 (88.8%)
Co-resident n (%)	12680 (47.2%)
Day Care n (%)	1152 (4.4%)
Home Care n (%)	8821 (32.5%)
Registration at memory clinic n (%)	19629 (68.4%)
Registration at primary care unit n (%)	9084 (31.6%)

**Fig 2:**
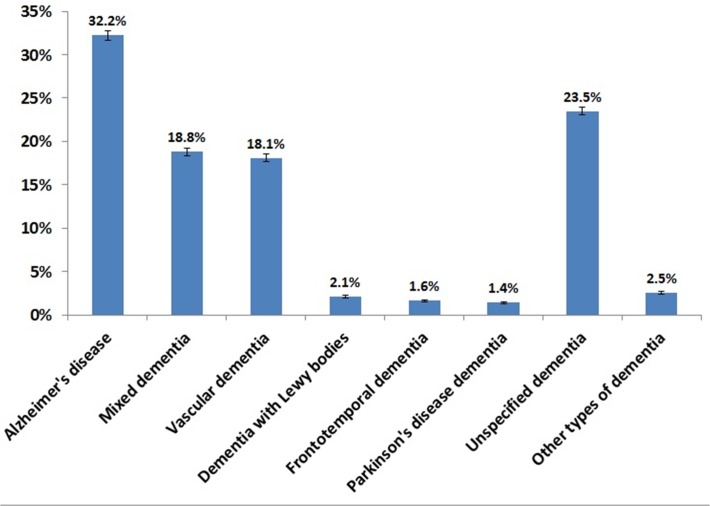
Frequency of different types of dementia in the whole population registered in SveDem during 2007–2012 (n = 28722).

Fig. 2 shows the proportion of different types of dementia disorders. The most common dementia disorder is AD (51%, including mixed dementia), while VaD accounted for about 18%. There were 23.5% of patients diagnosed with unspecified dementia. The number of newly diagnosed dementia patients who are entered into SveDem has been increasing. There were 805 individuals registered in 2007 and 3076 in 2008. The number of newly registered patients was 6994 in 2011 and 7280 in 2012. The mean age at the time of diagnosis steadily increased from 77.2 (SD = 8.3) years in 2007 to 80.3 (7.9) years in 2012, mainly due to a higher enrolment of patients from primary care centres.

### Coverage

**Fig 3:**
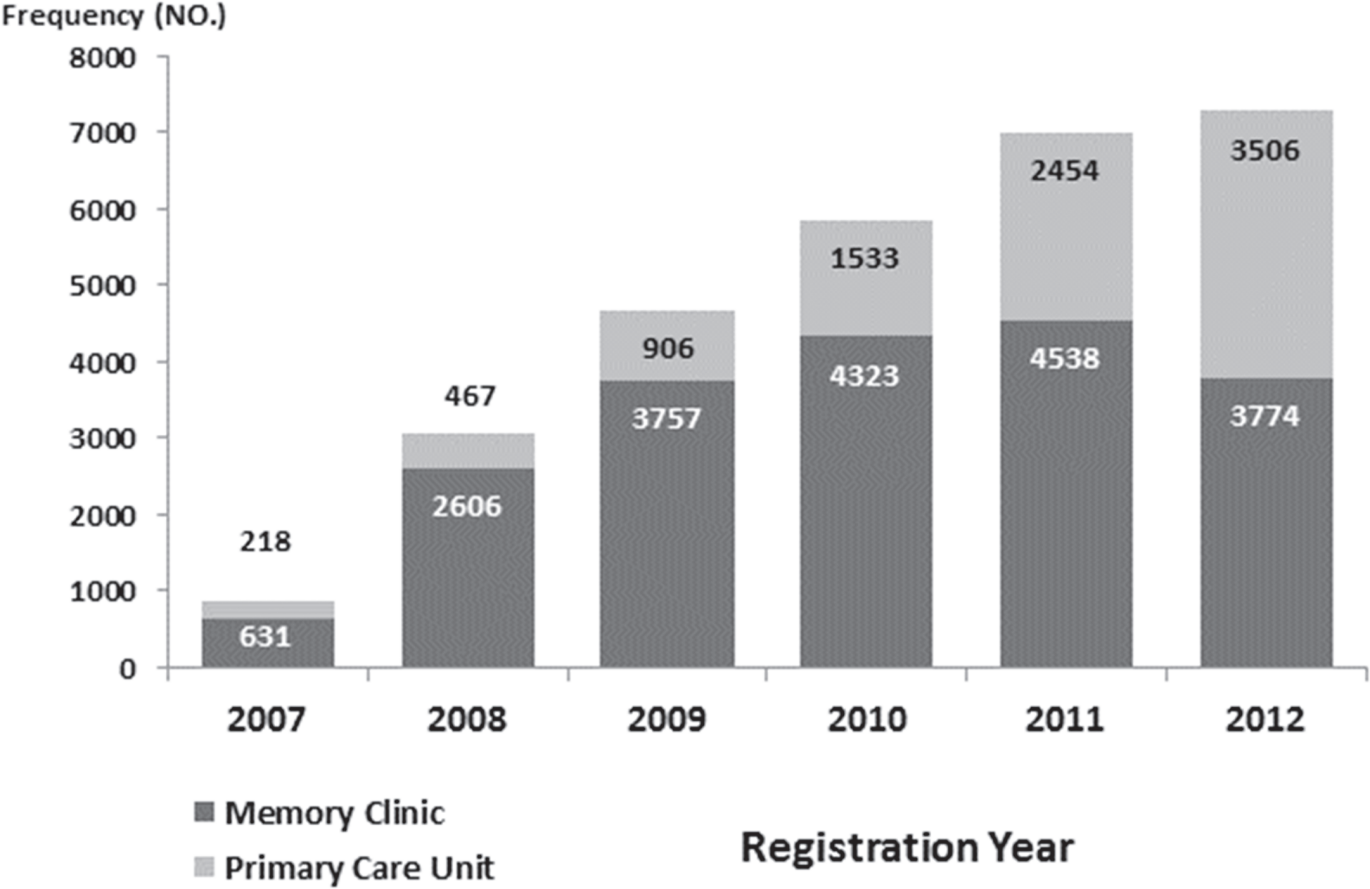
Frequency of dementia patients registered at specialist (memory clinic) and primary care units in SveDem during 2007–2012 (n = 28722).

SveDem started in 2007 when the majority of affiliated units were specialist settings, i.e. memory clinics. As illustrated in Fig. 3, the proportion of data from primary care units gradually increased from 25.6% in 2007 to 48.2% in 2012. In total, 19 629 (68.4%) dementia patients from memory clinics and 9084 (31.6%) patients from primary care units were registered. At the end of 2012, 58 specialist units (93% of all in Sweden) and 659 primary care centres (60% of all in Sweden) were affiliated to SveDem. Using an estimated incidence rate of 20.000 patients that develop dementia in Sweden each year [10], in relation to the registration of 7280 new patients in SveDem during 2012, an approximate coverage of incident dementia cases in SveDem in 2012 was 36%. During the 5-year period, 13 426 (46.7%) of all registered patients had at least one follow-up visit and 4427 (15.4%) patients were assessed more than twice.

### Quality indicators

In the recent publication of the Swedish National Guidelines for Care in cases of Dementia in 2010 [11], seven indicators were presented to evaluate the quality of dementia care: 1. Proportion of patients diagnosed with dementia during last year; 2. Proportion of patients undergoing basic dementia work-up; 3. Proportion of AD patients treated with cholinesterase-inhibitors and/or memantine; 4. Proportion of patients treated with antipsychotics in nursing homes; 5. Proportion of patients with day-care at diagnosis; 6. Proportion of patients living in nursing homes; 7. Proportion of patients followed-up at least once a year. For each indicator, SveDem has established internal goals to aim for. The results for 2011 and 2012 are shown in Table 3. Per these guidelines, the basic dementia work-up includes a structured clinical interview, an interview with a person close to the patient, an evaluation of the physical and psychological situation of the patient, cognitive testing with, at minimum, MMSE and clock test, cerebral imaging and blood analyses including calcium, homocysteine and thyroid function. The proportion of patients examined with a basic dementia work-up was 88% in 2012, not yet reaching the aim of 90%, while the proportion of AD patients who receive anti-dementia medication fulfils the goal of 80%. The goal to reduce the antipsychotic use in nursing homes to 10% has not been reached yet.

**Table 3:** Data from the SveDem population on quality indicators defined by the Swedish national guidelines for dementia care and treatment.

	Indicator	Year 2011	Year 2012
**1**	Proportion of patients diagnosed with dementia during last year	6994 (32.6)%	7280 (25.3%)
**2**	Proportion of patients undergoing basic dementia work-up*	6015 (86%)	6406 (88%)
**3**	Proportion of Alzheimer patients treated with cholinesterase-inhibitors or memantine	1763 (85%)	1641 (84%)
**4**	Proportion of patients treated with antipsychotics in nursing homes	136 (21.3%)	152 (18.5%)
**5**	Proportion of patients with day-care at diagnosis	252 (4.1%)	267 (4.1%)
**6**	Proportion of patients living in nursing homes	636 (9.1%)	823 (11.3%)
**7**	Proportion of patients followed-up at least once a year	4546 (65%)	3400 (46.7%)

* including patient history, blood tests, simple cognitive testing and CT (computed tomography)

## Discussion

SveDem is a quality registry that was initiated in 2007 to monitor and improve the quality of diagnostic work-up, treatment and care of dementia patients in Sweden. At the end of 2012 it included 28 722 newly diagnosed dementia patients who had a mean age of approximately 79 years and mean MMSE score of 21 at the time of registration. It is estimated that SveDem covers 36% of all incident dementia cases in Sweden.

There have been similar successful attempts in other countries to establish a quality dementia registry, such as in Denmark [12]. Whereas other registries have been developed to facilitate epidemiological studies [13], study the clinical expression of AD [14], provide a framework for recruitment of patients for clinical trials [15] or promote equality in the access for medical services [16].

SveDem aims to accomplish a similar high quality of dementia care in all regions in Sweden and in different clinical settings. Both specialist and primary care units are involved, making it possible to compare data from different care settings as well as follow patients through the health care system. The recruitment of memory clinics to SveDem is high (> 90%). In Sweden, dementia can be diagnosed either in memory clinics or in primary care centers, but patients are usually followed-up by general practitioners. However, younger patients, patients with uncommon diagnoses or complicating behavioural and psychological symptoms are referred to specialists. In order to ensure the adequate representativeness of the data from SveDem, the involvement of general practitioners is crucial. Therefore, there have been substantial efforts to implement SveDem in primary care in recent years.

There are differences in demographics between care within primary care and specialist care units. It was found that the mean age at diagnosis was higher in primary care than in specialist units. This may be due to the possibility that a younger person who shows symptoms of dementia will be referred to a specialist sooner because of uncharacteristic changes. Another difference found between primary and specialist care units in the yearly report in 2012 was that more advanced tests were performed within specialist units in order to determine the aetiology of the dementia disorder [17].

### Quality indicators

The results of quality indicator 1 (proportion of patients diagnosed with dementia within last year) must be judged with care. Firstly, the reference is the estimated incidence (based on epidemiology) while the registry covers newly diagnosed persons with dementia. Although “newly diagnosed” in most cases can be regarded as incident cases, it means that these persons for some reason have established contact with care, which in some cases may be in later stages than in the shift from mild cognitive impairment to manifest dementia. It also implies that the uncertainty in the epidemiological figures of incidence has not been taken into consideration. Furthermore, there is an unknown proportion of incident cases that do not have contact with the care system or if such contact exists, a diagnostic process has not yet been started. This unknown proportion is a part of the epidemiological based population of incident cases. Of great interest today is the promising indications of both lower age group related prevalence, and decreasing incidence of dementia [18–21]. Thus, the epidemiological figures may overestimate the present incidence of dementia and underestimate SveDem´s coverage.

Dementia disorders can be diagnosed in specialist or in primary care. The majority of the dementia patients diagnosed in specialist clinics are registered in SveDem and 93% of the specialist clinics are affiliated to SveDem, thus the coverage of patients from specialist units is very good. The coverage of dementia patients in primary care needs to be improved. A thorough implementation work has been painstakingly carried out during the last three years which have resulted in increased affiliation of primary care units and increased number of registered dementia patients.

The difficulty is to estimate 1. the number of patients who are not diagnosed at all and 2. the number diagnosed at each unit but not registered, ie “missed” patients. As denominator to estimate coverage, the overall dementia incidence of approximately 20 000 patients per year (data from the Swedish Board of Health and Welfare 2010) is often used, resulting in a coverage of 36% in 2012 (7280/20000). Patients who are not registered in SveDem probably do not differ from the patients in SveDem since the centres not affiliated to SveDem are similar to those already in the registry. SveDem works continually to increase the coverage and to increase the number of affiliated units (and patients) in the registry. The implementation work is carried out using different tools. Each region has a regional coordinator (usually a nurse) employed part-time to work with implementation of SveDem in recent years mainly focusing on primary care. The coordinators are responsible for information meetings for the staff of the regional primary care centers as well as conducting personal visits to units. There is also comprehensive information on the webpage (information on how to start SveDem at individual units and instruction films) and updates from the yearly national SveDem meetings, pdf-files of the yearly reports as well as documents on how to perform local quality work based on the unit´s SveDem data.

The data reported here for quality indicator 2 (proportion of patients undergoing basic dementia work-up—Table 3) is based on data from specialist centers and does not reflect the daily practice all over the country. As shown in the yearly SveDem report for 2012, the basic dementia work-up in primary care is lower [17]. The same is true for quality indicator 3 (proportion of AD patients treated with cholinesterase-inhibitors and/or memantine). The reported number of people that are treated with anti-dementia drugs is rather high. However, based on the national data on prescription and purchasing of these drugs, the figure for the whole country is probably lower, around 40% [22]. The proportion of patients treated with antipsychotics in nursing homes (indicator 4) is regarded as too high [23,24], as antipsychotic medications have been associated with cardio- and cerebrovascular events and mortality in older people [25–27]. Therefore, the Swedish guidelines for the treatment of neuropsychiatric symptoms in dementia state that antipsychotic medication should be used very restrictively and only for psychotic symptoms or aggression that causes suffering or potential danger to the patient or others [19]. However, compared to several other countries, the number of dementia patients treated with antipsychotic medications is rather low in Sweden [28–30].

The proportion of people with dementia with day-care and those who live in nursing homes (indicators 5 and 6) is rather low since the database, so far, consists of people in the early stages of dementia. For the whole dementia population it is estimated that about 45% live in nursing homes or other sorts of sheltered living [31]. The follow-ups in SveDem (indicator 7) offer great opportunities to analyse the dynamics of dementia as the course of the disease continues. However, at this time, there is not sufficient data that allows us to conclude how well the follow-ups are conducted.

### Dementia diagnosis

The data in SveDem represents the diagnoses of dementia made in a clinical setting and the accuracy of the diagnosis has not been examined. Therefore, the registry is limited by the fact that neurodegenerative changes often overlap with vascular pathology and the border between AD, mixed dementia and vascular dementia is often subtle. The overall proportion of mixed dementia is rather high in SveDem (about 19%), but the reason for this needs further analyzing. However, the proportion of AD including mixed dementia is 51% and VaD 18%, which is similar or little lower than the reported proportion of AD and VaD in other cohorts [32]. However, the proportion of unspecified dementia is high and probably includes a number of AD and VaD patients. Epidemiological studies show that both AD and VaD share similar risk factors [33] and vascular pathology has been observed in autopsy investigations on AD patients more frequently than expected [34]. There has been a lack of consensus on integrating vascular changes into diagnostic criteria of dementia [34], therefore the diagnosis of different dementia disorders may vary in several clinical centers. Furthermore, misclassification between PDD and DLB is common [35] as these disorders often overlap in pathology and clinical manifestation [36]. On the other hand, the true incidence of PDD and DLB is uncertain and therefore the increasing number of dementia patients in SveDem may with time contribute with more accurate incidence numbers of DLB and PDD.

A clear difference between primary care and specialist care is the higher proportion of unspecified dementia among patients diagnosed by primary care physicians. There may be several reasons for this discrepancy, but the obvious explanation is that some patients are too frail to undergo a thorough diagnostic process or present diagnostic difficulties and receive a diagnosis of “unspecified dementia” pending a referral to specialist care. But there is also a number of patients who would benefit from obtaining a specific dementia diagnosis. Indeed, SveDem data shows that the proportion of unspecified dementia diagnosis in primary care is decreasing [37], suggesting that initiatives to help to structure the dementia care have a positive effect.

### Patient reported outcome measure

There is an ongoing discussion in Swedish quality registries about how to involve patients´ own evaluations on their health, i.e. patient reported outcome measure (PROM). In a Swedish rheumatoid arthritis registry, PROM is included in the form of patients´ reports on their joint conditions, pain and quality of life [38]. We have recently included “The Quality of Life in Late-Stage Dementia” (QUALID) [39] into our registry, which is a validated assessment of dementia patients by care givers suitable for patients in advanced dementia stages in nursing homes. However, there is a need to evaluate the quality of life of dementia patients also in mild to moderate stages. The decline in cognitive functioning represents a major challenge for the introduction of PROM into SveDem. Patient and proxy-reported health utility and quality of life may differ [40], thus it is important to include opinions of both patients and caregivers when evaluating the quality of life.

### Future perspectives

Even though SveDem was established primarily as a quality registry, it can offer many opportunities for research and function as a framework for a wide range of studies. The first scientific paper based on SveDem was published in 2012 and focused on the diagnosis of dementia in clinical settings [41]. Since then there have been thirteen more studies published [36,42–53]. In the future, the comparison of national results regarding dementia care and treatment with data from similar databases in other countries can help identify differences in dementia care internationally. Linking SveDem with other quality registries in Sweden can help answer questions regarding the treatment of dementia patients affected with e.g. diabetes, hip fractures or heart failure and create new research hypotheses. Furthermore, SveDem could provide a solid base for health economic studies that could aid political decision makers. SveDem’s data is available to researchers who are encouraged to establish contact with the registry holder or the steering committee to access the data.

Research on dementia is often performed on individuals who do not reflect the population at risk because the oldest old are excluded from many clinical trials [54]. SveDem could address these methodological problems and function as a tool to identify and recruit patients that are suitable for studies. Given the possibility to observe the clinical course and progression of dementia, SveDem can facilitate certain improvements in designing clinical trials. The registry could also serve to evaluate new interventions and therapeutic options and determine their impact on outcomes to help further develop national or international guidelines. In addition, SveDem could play an essential role in monitoring changes in a diagnostic approach towards dementia with regard to the newly proposed diagnostic criteria [55,56].

## Conclusion

SveDem is a quality registry that aims to monitor and improve the diagnostic work-up, treatment and care of patients with dementia. SveDem can coordinate a collaboration between patients, clinicians, researchers and carers and create a large network that addresses current needs in the dementia field. It can contribute to the enhancement of expertise and ensure that up-to-date research becomes embedded in clinical practice.

## Supporting Information

S1 Table Variables collected in nursing homes. (DOCX)
