# Supplementary material for: SveDem, the Swedish Dementia Registry – A Tool for Improving the Quality of Diagnostics, Treatment and Care of Dementia Patients in Clinical Practice
Source: PLoS One. 2015 Feb 19;10(2):e0116538. doi: 10.1371/journal.pone.0116538 (PMC4335024; doi:10.1371/journal.pone.0116538)
Supplement: S1 Table — (DOCX) [file pone.0116538.s001.docx]

**Table S1. Variables collected in nursing homes**

| Social security number |
| --- |
| Type of dementia |
| Date of diagnosis |
| Date of follow-up |
| Date of moving to nursing home |
| Type of nursing home |
| Weight |
| Mini Mental State Exam score |
| **Diagnosis** |
| Change of diagnosis |
| **Medications at follow-up** |
| Number of drugs patient continuously treated with at diagnosis |
| Cholinesterase inhibitors |
| N-methyl-D-aspartate receptor antagonists |
| Cardiovascular medication |
| Antidepressants |
| Neuroleptics |
| Anxiolytics |
| Hypnotics |
| Additional medication for dementia |
| Structured medication follow up |
| **Function Assessment Measures: Alzheimer’s Disease Co-operative Study - Activities of Daily Living Inventory** |
| Regarding eating |
| Regarding walking |
| Regarding bowel and bladder function at the toilet |
| Regarding bathing |
| Regarding grooming |
| Regarding dressing |
| **Care interventions** |
| Structured screening for: |
| Fall |
| Decubitus ulcers |
| Malnutrition |
| Oral health |
| Structured intervention for: |
| Fall |
| Decubitus ulcers |
| Malnutrition |
| Oral health |
| **Person-centred care** |
| Patient’s narrative |
| Mutual care plan and documentation |
| Individual attitude |
| Individual protection measures |
| **Quality of life (Qalid)** |
| Quality of life in severe dementia Points |
